# Endovascular treatment with aortic endoprostheses for subclavian artery aneurysm secondary to late traumatic axillary-axillary arteriovenous fistula

**DOI:** 10.1590/1677-5449.210016

**Published:** 2022-01-07

**Authors:** Vinicius Tadeu Ramos da Silva Grillo, Rodrigo Gibin Jaldin, Felipe Damascena Rosa, Mariana Thais Silva Secondo, Rafael Elias Farres Pimenta, Matheus Bertanha, Marcone Lima Sobreira, Winston Bonetti Yoshida

**Affiliations:** 1 Universidade Estadual “Júlio de Mesquita Filho” – UNESP, Faculdade de Medicina de Botucatu, Hospital das Clínicas, Botucatu, SP, Brasil.

**Keywords:** arteriovenous fistula, subclavian artery, vascular system injuries, endovascular procedures

## Abstract

Traumatic arteriovenous fistulas (AVFs) involving the axillary and subclavian vessels are uncommon and account for 5 to 10% of all arterial traumas. The complex anatomy of this region makes treatment of this segment challenging. In this therapeutic challenge, we describe the case of a 73-year-old man, referred for progressive edema and ulceration involving the right upper limb and with a history of gunshot wound to the right infraclavicular region about 50 years previously. Angiotomography was performed and an axillary-axillary AVF was found, associated with tortuosity and aneurysmatic dilation of the subclavian artery downstream. He underwent endovascular intervention and a conical (monoiliac) 26 × 14 × 90 mm Braile® endoprosthesis was used in the aneurysmatic subclavian artery, posterior to the exit of the right vertebral artery and a 16 × 16 × 95mm Excluder® monoiliac endoprosthesis was placed overlapping the first prosthesis, showing a satisfactory result. Therefore, the possibility of successfully using aortic endoprostheses in an unusual and exceptional situation is described.

## INTRODUCTION

Traumatic arteriovenous fistulas (AVFs) involving the axillary vessels are uncommon,^1^ as are those in the subclavian region,^2^^,^^3^ and AVFs in these areas account for 5 to 10% of arterial traumas in civilians.^4^ The principal etiology is penetrating trauma,^3^ and gunshot wounds are associated with 65.2% of cases, while the most prevalent injuries observed were pseudoaneurysm and AVF.^5^^,^^6^ Although these events are uncommon, they are associated with elevated morbidity and mortality,^3^^,^^7^ with high rates of in-hospital death, primarily caused by hemorrhagic shock.^1^

When these vascular injuries are not diagnosed at the time of trauma, patients can remain asymptomatic for years before clinical manifestations of AVFs are identified: continuous thrills and murmurs with systolic accentuation in the topography of the vessels involved, collateral venous circulation, heart failure, reduced perfusion of the ipsilateral limb, and signs of venous hypertension such as edema, which can progress to ulceration and gangrene in advanced cases.^1^^,^^2^^,^^7^^-^9

The complex anatomy of this region and the clinical severity these patients are factors that elevate morbidity and mortality rates,^1^^,^^3^ making treatment of this segment a challenge for the surgeon.^10^ This study was duly analyzed and approved by a Research Ethics Committee (CAAE 45890921.0.0000.5411, ruling number 4.699.405).

### Part I – clinical situation

**Figure 1:**
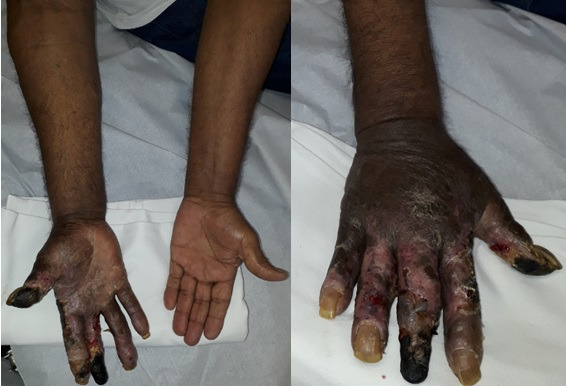
Initial appearance of the lesions at the time of admission. Note the edema of the right upper limb, with darkening of the hand and forearm and trophic ulcers (lesions).

A 73-year-old man was referred because of progressive edema of the right upper limb (RUL) associated with a venous stasis ulcer on the right hand with onset 6 months previously. He had a history of arterial hypertension, was taking losartan, and had no other comorbidities, but reported having suffered a gunshot wound to the infraclavicular region around 50 years previously which, at the time, had not had any major repercussions, or been subjected to surgical or diagnostic interventions. The patient had noticed progressive emergence of varicose veins in the area of the projectile entry wound, in the right infraclavicular region, which had begun to appear after the trauma and increased in number and caliber as the years passed. Physical examination identified edema of the shoulder, forearm, and right hand and varicose veins extending from the forearm to the infraclavicular region, a darkened hand, areas of dry necrosis involving the 1st and 3rd fingers and a large area of ulceration on the dorsal aspect of the hand (Figure 1). The left upper limb and both lower limbs were free from edema and trophic ulcers (lesions). Radial, ulnar, and distal lower limb pulses were palpable and strong.

**Figure 2:**
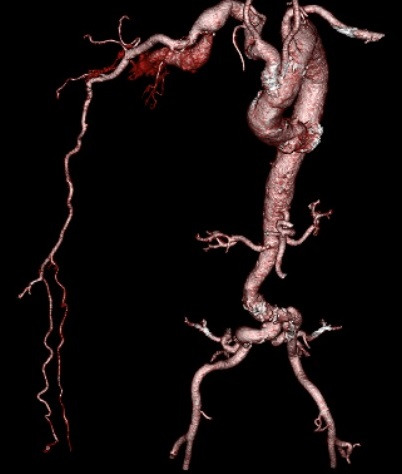
Three-dimensional reconstruction of the angiotomography, showing a right axillary-axillary AVF associated with tortuosity and aneurysm of the right subclavian artery downstream.

In the right infraclavicular topography, auscultation detected a continuous murmur with systolic accentuation. Laboratory tests requested at admission, including hemogram, platelets, renal function tests, creatine phosphokinase (CPK), coagulogram, and electrolytes, were all normal. The patient underwent angiotomography of the thorax, abdomen, and RUL (Figure 2), which identified an axillary-axillary AVF associated with tortuosity and aneurysmal dilatation of the subclavian artery downstream, with a greatest diameter of 24 mm and length of 125 mm.

If surgery is indicated, treatment would be:

- Conventional open surgery, with thoracotomy and ligature of the AVF or aneurysmectomy with arterial bypass;
- Endovascular treatment with embolization of the AVF with a vascular plug; or
- Endovascular treatment and off-label deployment of an aortic endoprosthesis, due to the diameter and length of the subclavian artery.

### Part II – what was done

**Figure 3:**
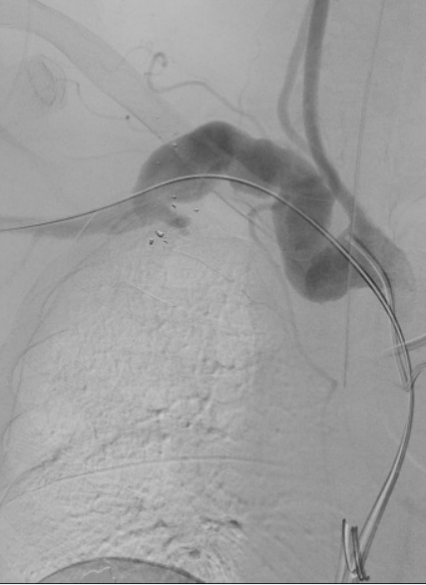
Digital subtraction angiography showing the tortuous right subclavian artery with aneurysmal dilatation. Fragments of the firearm projectile can be seen in the topography of the injury.

**Figure 4:**
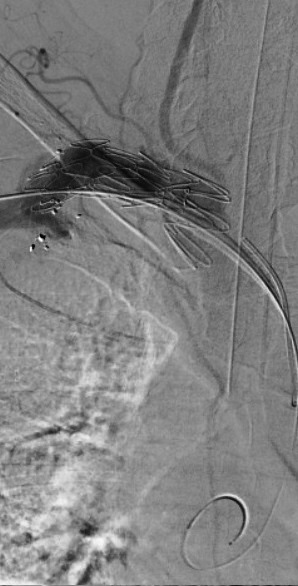
Digital subtraction angiography showing the 26 × 14 × 90 mm Braile® monoiliac endoprosthesis (Braile Biomédica, São José do Rio Preto, Brazil) positioned in the aneurysmal subclavian artery, posterior to the origin of the right vertebral artery, which was maintained patent by the free-flow graft.

**Figure 5:**
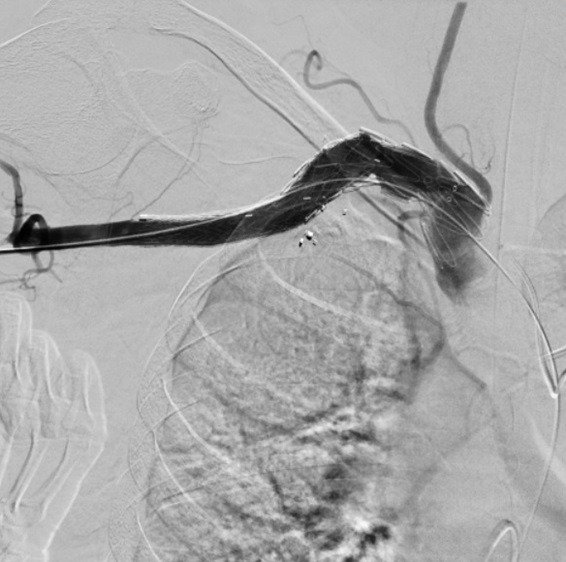
Digital subtraction angiography showing the 16 × 16 × 95 mm monoliliac Excluder® endoprosthesis (WL Gore & Associates, Flagstaff, United States) positioned overlapping the first prosthesis. Note the preservation of the right vertebral artery.

Endovascular intervention was chosen to avoid thoracotomy. A longitudinal incision was made on the medial surface of the proximal third of the right arm and the brachial artery (RBA) was identified. The axillary-brachial transition was punctured under direct view (the axillary artery diameter was approximately 6 mm) and a 6F introducer was inserted. The lesion was crossed with a 0.035” Stiff Roadrunner® (Cook Medical, Bloomington, Ind) guidewire and a 5F vertebral catheter, positioned in the ascending aorta. The guidewire was then exchanged for a Lunderquist® Super-stiff (Cook Medical, Bloomington, Ind) and the vertebral catheter was removed. The brachiocephalic trunk was catheterized with a Simmons 2 (Cook Medical, Bloomington, Ind) catheter over a 0.035” Stiff Roadrunner® (Cook Medical, Bloomington, Ind) guidewire (Figure 3), using retrograde puncture of the right common femoral artery and insertion of a 5F introducer. Via the RUL access, a 26 × 14 × 90 mm conical (monoiliac) 18Fr Braile® endoprosthesis (Braile Biomédica, São José do Rio Preto, Brazil) was deployed into the aneurysmal subclavian artery, posterior to the origin of the right vertebral artery (RVA) (Figure 4). Next, a 16 × 16 × 95 mm Excluder® bifurcated endoprosthesis branch extension (WL Gore & Associates, Flagstaff, United States) was released (Figure 5), overlapping the first prosthesis. A type 3 endoleak was seen on control imaging and was fully resolved by ballooning with an Equalizer balloon, achieving immediate sealing of the AVF, treatment of the aneurysmal dilatation of the subclavian artery, and maintenance of RVA patency via the free-flow endoprosthesis.

**Figure 6:**
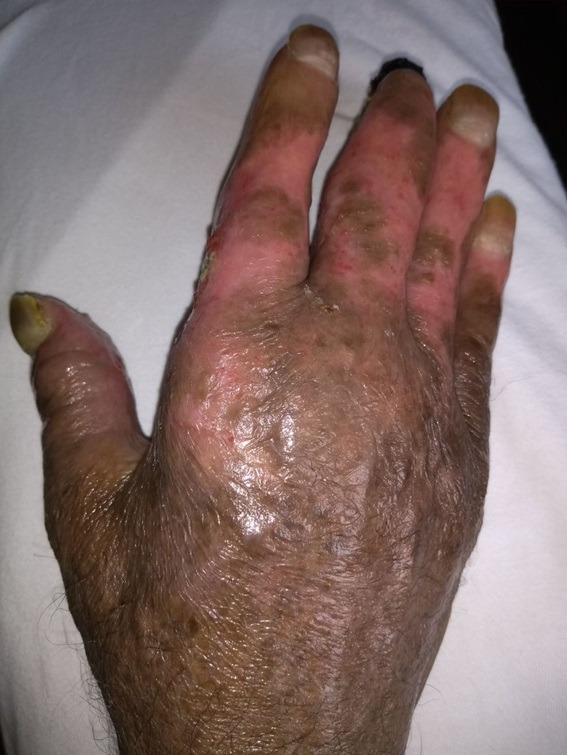
Appearance of the right hand after 30 days, with healed trophic ulcers and necrosis only remaining at the distal phalanx of the third finger.

**Figure 7:**
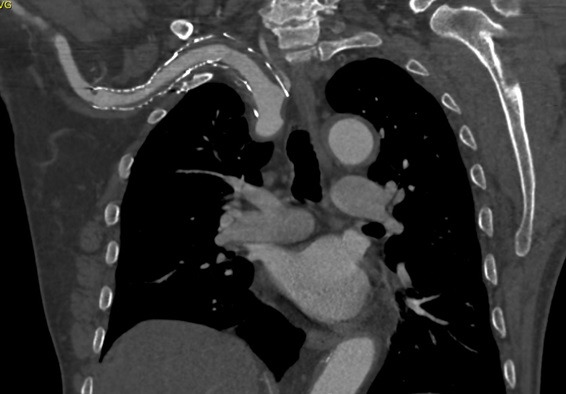
Coronal slice from the control angiotomography at 10 months, showing patent endoprostheses with no endoleaks. Note that the right vertebral artery is patent.

On the third day after endovascular treatment, the patient underwent debridement of the necrotic tissues and was discharged from hospital after 7 days with a prescription for ciprofloxacin and clindamycin to complete the 14-day cycle, plus dual anti-platelet therapy with acetylsalicylic acid (ASA) 100 mg/day and clopidogrel 75 mg/day and medium compression elastic bandaging (20-30 mmHg) on the RUL. At the 30-day outpatients follow-up visit, the edema and signs of venous hypertension had reduced and the trophic ulcers had healed, with the only remaining necrosis involving the distal phalanx of the third finger, which was amputated (Figure 6). At a 10-month postoperative follow-up assessment, vascular echography with Doppler of the arteries showed that the endoprostheses were patent and free from hemodynamically significant stenosis, while angiotomography confirmed the normal ultrasound findings (Figure 7). At the latest outpatients assessment, 25 months after the operation, the patient was free from complaints, his wounds were healed, he had no residual edema, and his radial and ulnar pulses were palpable.

## DISCUSSION

Conventional surgical treatment of lesions in the topography of the subclavian and axillary vessels is complex, very often requiring supra and/or infraclavicular incisions and, for proximal control, a midline sternotomy may be needed to access the right subclavian artery, or an anterolateral thoracotomy over the third or fourth intercostal space on the left.^5^^,^^6^^,^^10^

In view of this, endovascular treatment is an attractive option. Possible treatments for traumatic AVFs include covered stents or peripheral endoprostheses,^2^^,^^5^^,^^6^^,^^8^^,^^11^^,^^12^ with shorter operating times and less blood loss.^1^

In this particular case, it was the authors’ opinion that the best option was to use an aortic endoprosthesis, because the conventional open surgery option is associated with greater morbidity and mortality. The possibility of using a covered stent was not considered because of the diameter and length of the subclavian artery aneurysm, since the materials available at the time were of limited diameter and length. Embolization with a vascular plug could have been sufficient to treat the AVF, but, because of the diameter of the aneurysm, the patient could have developed intraluminal thrombi and consequent distal embolization, and the aneurysm diameter could have increased, compressing adjacent structures or leading to rupture.

In addition to the success of the surgery, pulmonary hypertension can be corrected and symptoms related to AVF resolved, such as secondary congestive heart failure, even severe forms.^9^ The long-term durability of endoprostheses in this segment has not yet been established and, as a result, there is no consensus on routine use of endovascular treatment.^2^ A recent study assessing endovascular treatment for axillary-subclavian injuries found intrahospital mortality of 5.4% and primary stent patency of 88% over a mean period of 13.2 months.^5^

Upper limb vascular traumas leading to AVF formation are rare, have insidious course, and are difficult to diagnose in the initial phases. Endovascular treatment with covered stents is safe and can be employed. However, in the case described, the diameter and length of the aneurysm precluded their use. Therefore, we present the possibility of using an aortic endoprosthesis in an uncommon and exceptional situation to treat an upper limb AVF, with therapeutic success.

## References

[1] Mo A (2014). Endovascular repair of traumatic arteriovenous fistula between axillary artery and vein. Chin J Traumatol.

[2] Maués JJB, Hauter HL (2018). Tratamento endovascular de fístula traumática de vasos subclávios: relato de caso. J Vasc Bras.

[3] Cohen JE, Rajz G, Gomori JM (2008). Urgent endovascular stent-graft placement for traumatic penetrating subclavian artery injuries. J Neurol Sci.

[4] Castelli P, Caronno R, Piffaretti G (2005). Endovascular repair of traumatic injuries of the subclavian and axillary arteries. Injury.

[5] Branco BC, DuBose JJ (2016). Endovascular solutions for the management of penetrating trauma: an update on REBOA and axillo-subclavian injuries. Eur J Trauma Emerg Surg.

[6] Jaldin RG, Bertanha M, Sobreira ML (2013). pseudoaneurisma da artéria subclávia próximo à origem da artéria vertebral após punção inadvertida: Tratamento endovascular ou cirurgia aberta?. J Vasc Bras.

[7] Oliveira PPM, Petrucci O, Vilarinho KADS, Silveira LM, Vieira RW, Braile DM (2008). Fístula traumática entre tronco braquiocefálico e veia braquiocefálica por arma de fogo. Arq Bras Cardiol.

[8] Santos EP, Batista RRA, Felici FM, Correia VE, Oliveira MB, Alves RF (2014). Endovascular correction of a traumatic internal iliac arteriovenous fistula with a covered stent. J Vasc Bras.

[9] Pilan BF, Oliveira AM, Siqueira DED, Guillaumon AT (2014). Tratamento de fístula arteriovenosa adquirida com repercussões hemodinâmicas graves: desafio terapêutico. J Vasc Bras.

[10] Medeiros CAF, Landim RM, Castro AN (2003). Condutas no trauma penetrante da artéria axilar. J Vasc Bras.

[11] Ramacciotti E, Gerardi VA, Fagundes DJ (1999). Endovascular de Fístulas Artériovenosas. Acta Cir Bras.

[12] Medeiros CAF, Hatsumura TC, Gusmão DR, Freire LMD, Rocha EF, Guillaumon AT (2008). Tratamento endovascular do trauma arterial dos membros. J Vasc Bras.

