# Supplementary material for: Endovascular treatment with aortic endoprostheses for subclavian artery aneurysm secondary to late traumatic axillary-axillary arteriovenous fistula
Source: J Vasc Bras. 2022 Jan 7;20:e20210016. doi: 10.1590/1677-5449.210016 (PMC8759580; doi:10.1590/1677-5449.210016)
Supplement: Translation

# Translation: Tratamento endovascular com endoprótese aórtica para aneurisma de artéria subclávia secundário à fístula arteriovenosa axilo-axilar traumática tardia

**Authors:** Vinicius Tadeu Ramos da Silva Grillo, Rodrigo Gibin Jaldin, Felipe Damascena Rosa, Mariana Thais Silva Secondo, Rafael Elias Farres Pimenta, Matheus Bertanha, Marcone Lima Sobreira, Winston Bonetti Yoshida

**Affiliations:** 1 Universidade Estadual “Júlio de Mesquita Filho” – UNESP, Faculdade de Medicina de Botucatu, Hospital das Clínicas, Botucatu, SP, Brasil.

**Keywords:** fístula arteriovenosa, artéria subclávia, lesões do sistema vascular, procedimentos endovasculares

## Abstract

As fístulas arteriovenosas (FAVs) traumáticas envolvendo os vasos axilares e subclávios são incomuns e correspondem de 5 a 10% de todos os traumas arteriais. A anatomia complexa dessa região torna desafiador o tratamento desse segmento. Neste desafio terapêutico, descrevemos o caso de um homem de 73 anos, encaminhado por edema progressivo e úlcera no membro superior direito, com história pregressa de ferimento por arma de fogo na região infraclavicular direita há cerca de 50 anos. Foi realizada angiotomografia e identificou-se FAV axilo-axilar associada à tortuosidade e dilatação aneurismática de artéria subclávia a jusante. O paciente foi submetido à intervenção endovascular com endoprótese cônica (monoilíaca) 26 × 14 × 90 mm Braile® na artéria subclávia aneurismática, posterior à saída da artéria vertebral direita, e endoprótese monoilíaca 16 × 16 × 95 mm Excluder® com sobreposição na primeira prótese, apresentando resultado satisfatório. Portanto, descreve-se a possibilidade de utilização de endoprótese aórtica em situação incomum e de exceção, com sucesso.

## INTRODUÇÃO

As fístulas arteriovenosas (FAVs) traumáticas envolvendo os vasos axilares são incomuns^1^, assim como no território subclávio^2^^,^^3^, e as FAVs nessas regiões correspondem de 5 a 10% dos traumas arteriais em civis^4^. A principal etiologia é o trauma penetrante^3^, sendo o ferimento por arma de fogo associado em 65,2% dos casos, e as lesões mais comumente encontradas foram pseudoaneurisma e FAV^5^^,^^6^. Apesar de incomuns, associam-se a elevada morbimortalidade^3^^,^^7^, com altas taxas de óbito ainda no ambiente pré-hospitalar, principalmente devido a choque hemorrágico^1^.

Quando essas lesões vasculares não são diagnosticadas no momento do trauma, os pacientes podem evoluir assintomáticos por anos até que as manifestações clínicas das FAVs sejam identificadas: sopros e frêmitos contínuos com reforço sistólico na topografia dos vasos, circulação colateral venosa, insuficiência cardíaca, diminuição da perfusão do membro ipsilateral e sinais de hipertensão venosa como edema, podendo chegar a ulcerações e gangrena em casos avançados^1^^,^^2^^,^^7^^-^9.

A anatomia complexa dessa região e a gravidade clínica do paciente são fatores que aumentam as taxas de morbimortalidade^1^^,^^3^, tornando desafiador o tratamento desse segmento para o cirurgião^10^. Este estudo foi devidamente avaliado e aprovado pelo Comitê de Ética em Pesquisa (CAAE 45890921.0.0000.5411, parecer número 4.699.405).

### Parte I – situação clínica

Um homem de 73 anos foi encaminhado por história de edema progressivo no membro superior direito (MSD) associado à úlcera de estase venosa na mão direita há 6 meses. Apresentava antecedente de hipertensão arterial, em uso de losartana, sem outras comorbidades, e relatou história pregressa de ferimento por arma de fogo na região infraclavicular direita há cerca de 50 anos, à época sem maiores repercussões, intervenções cirúrgicas ou diagnósticas. O paciente notou aparecimento progressivo de veias varicosas na topografia do orifício de entrada do projétil na região infraclavicular direita, iniciado após o trauma e que aumentou em número e calibre no decorrer dos anos. Ao exame físico, apresentava-se com edema de ombro, antebraço e mão direita, associado a veias varicosas desde antebraço até região infraclavicular, escurecimento da mão, áreas de necrose seca no 1º e 3º quirodáctilos e grande ulceração em face dorsal da mão (Figura 1). O membro superior esquerdo e os membros inferiores bilateralmente não apresentavam edema ou lesões tróficas. Os pulsos radiais, ulnares e distais de membros inferiores eram palpáveis e amplos.

**Figura 1:** Aspecto inicial das lesões no momento da admissão. Nota-se edema de membro superior direito associado a escurecimento da mão e antebraço e lesões tróficas.

Na topografia infraclavicular direita, auscultava-se sopro contínuo com reforço sistólico. Quanto aos exames laboratoriais de entrada, hemograma, plaquetas, função renal, creatinofosfoquinase (CPK), coagulograma e eletrólitos estavam normais. O paciente foi submetido à angiotomografia de tórax, abdômen e MSD (Figura 2), sendo identificada FAV axilo-axilar associada à tortuosidade e dilatação aneurismática de artéria subclávia a jusante, com maior diâmetro de 24 mm e extensão 125 mm.

**Figura 2:** Reconstrução tridimensional da angiotomografia, evidenciando FAV axilo-axilar direita associada à tortuosidade e aneurisma de artéria subclávia direita a jusante.

Caso indicada a abordagem cirúrgica, as opções de tratamento seriam:

- Cirurgia aberta convencional, com toracotomia e ligadura da FAV ou aneurismectomia com derivação arterial;
- Abordagem endovascular e embolização da FAV com plugue vascular;
- Abordagem endovascular e utilização *off-label* de endoprótese aórtica devido ao diâmetro e à extensão da artéria subclávia.

### Parte II – o que foi feito

Foi decidido pela intervenção endovascular para evitar toracotomia. Realizou-se incisão longitudinal na face medial do terço proximal do braço direito e reparo da artéria braquial (ABD). Foi realizada punção da transição axilo-braquial sob visão direta (diâmetro da artéria axilar de aproximadamente 6 mm) e passagem do introdutor 6F, transposição da lesão com fio guia 0,035” Stiff Roadrunner® (Cook Medical, Bloomington, Ind) sob cateter vertebral 5F, posicionando-o na aorta ascendente. Trocou-se o fio guia pelo Super-stiff Lunderquist® (Cook Medical, Bloomington, Ind), e foi retirado o cateter vertebral. Por punção retrógrada de artéria femoral comum direita e passagem do introdutor 5F, foi cateterizado o tronco braquiocefálico com fio guia 0,035” Stiff Roadrunner® (Cook Medical, Bloomington, Ind) sob cateter Simmons 2 (Cook Medical, Bloomington, Ind) (Figura 3). Na via MSD, foi feito implante de endoprótese cônica (monoilíaca) 26 × 14 × 90 mm de perfil 18Fr Braile® (Braile Biomédica, São José do Rio Preto, Brasil) na artéria subclávia aneurismática, posterior à saída da artéria vertebral direita (AVD) (Figura 4). Na sequência, foi liberada uma extensão de ramo de endoprótese bifurcada de 16 × 16 × 95 mm Excluder® (WL Gore & Associates, Flagstaff, EUA) (Figura 5) com sobreposição na primeira prótese. Evidenciou-se *endoleak* tipo 3 na imagem de controle, sendo totalmente tratado por balonamento com balão Equalizer, com selamento imediato da FAV, tratamento de dilatação aneurismática de artéria subclávia e manutenção da perviedade da AVD pelo *free flow* da endoprótese.

**Figura 3:** Angiografia por subtração digital evidenciando artéria subclávia direita tortuosa e com dilatação aneurismática. Nota-se estilhaços do projétil de arma de fogo na topografia da lesão.

**Figura 4:** Angiografia por subtração digital evidenciando posicionamento de endoprótese monoilíaca 26 × 14 × 90 mm Braile® (Braile Biomédica, São José do Rio Preto, Brasil) em artéria subclávia aneurismática, posterior à saída da artéria vertebral direita que foi mantida pérvia pelo *free flow*.

**Figura 5:** Angiografia por subtração digital evidenciando posicionamento de endoprótese monoilíaca 16 × 16 × 95 mm Excluder® (WL Gore & Associates, Flagstaff, EUA) com sobreposição na primeira prótese. Nota-se a manutenção da artéria vertebral direita.

No terceiro dia após a abordagem endovascular, o paciente foi submetido ao desbridamento de tecidos desvitalizados e recebeu alta hospitalar após 7 dias com prescrição de ciprofloxacino e clindamicina para completar o ciclo de 14 dias, além de dupla antiagregação com ácido acetilsalicílico (AAS) 100 mg/dia, clopidogrel 75 mg/dia e faixa elástica de média compressão (20-30 mmHg) no MSD. No retorno ambulatorial de 30 dias, observou-se diminuição do edema e dos sinais de hipertensão venosa, além de cicatrização das lesões tróficas, mantendo apenas a delimitação da necrose em falange distal do 3º quirodáctilo, a qual foi amputada (Figura 6). Na avaliação com 10 meses de pós-operatório, foi realizada ecografia vascular com Doppler (EVD) arterial e evidenciado as endopróteses pérvias e sem estenoses hemodinamicamente significativas, além de angiotomografia confirmando achados de normalidade do ultrassom (Figura 7). Em última avaliação ambulatorial com 25 meses de pós-operatório, o paciente apresentava-se sem queixas, com feridas cicatrizadas, sem edema residual e com pulsos radial e ulnar palpáveis.

**Figura 6:** Aspecto da mão direita após 30 dias, evidenciando cicatrização das lesões tróficas, mantendo apenas necrose em falange distal do 3º quirodáctilo.

**Figura 7:** Corte coronal de angiotomografia de controle após 10 meses, evidenciando endopróteses pérvias e sem *endoleaks*. Nota-se artéria vertebral direita pérvia.

## DISCUSSÃO

O tratamento cirúrgico convencional para lesões no território dos vasos subclávios e axilares é complexo, muitas vezes envolvendo incisão supra e/ou infraclavicular, e, para controle proximal, pode ser necessário esternotomia mediana para acessar a artéria subclávia direita ou toracotomia anterolateral no 3º ou 4º espaço intercostal para a esquerda^5^^,^^6^^,^^10^.

Diante disso, o tratamento endovascular torna-se opção atraente. Para o tratamento das FAVs traumáticas, há a possibilidade de utilização de stents recobertos ou endopróteses periféricas^2^^,^^5^^,^^6^^,^^8^^,^^11^^,^^12^, com menor tempo cirúrgico e menor perda sanguínea^1^.

Particularmente, neste caso, a utilização da endoprótese aórtica foi a melhor opção na opinião dos autores, pois a abordagem cirúrgica aberta convencional está relacionada com maior morbimortalidade. Não foi aventada a possibilidade de utilização do stent revestido devido ao diâmetro e comprimento do aneurisma da artéria subclávia, pois os materiais disponíveis até o momento apresentam diâmetros e extensão limitados. A embolização com plugue vascular poderia ser suficiente para o tratamento da FAV, porém, devido ao diâmetro do aneurisma, o paciente poderia evoluir com trombos intraluminais e consequente embolização distal, além do aumento do diâmetro e compressão de estruturas adjacentes ou rotura do aneurisma.

Além do sucesso cirúrgico, cita-se a correção da hipertensão pulmonar e dos sintomas relacionados à FAV, como a insuficiência cardíaca congestiva secundária, mesmo em forma grave^9^. A longo prazo, ainda não se estabeleceu a durabilidade de próteses endovasculares nesse segmento; portanto, não há consenso do uso rotineiro do tratamento endovascular^2^. Em estudo recente avaliando o tratamento endovascular para lesões axilo-subclávias, foi encontrada mortalidade intra-hospitalar de 5,4% e patência primária do stent de 88% no período médio de 13,2 meses^5^.

Os traumas vasculares da extremidade superior com formação de FAV são raros, de curso insidioso e de difícil diagnóstico na sua fase inicial. A abordagem endovascular com uso de stents revestidos é segura, podendo ser empregada. No entanto, no caso relatado, o diâmetro e comprimento do aneurisma limitaram seu uso. Portanto, apresentamos a possibilidade de utilização de endoprótese aórtica em situação incomum e de exceção para tratamento de FAV na extremidade superior, com sucesso terapêutico.
